# Tooth Wear in Older Adults: A Review of Clinical Studies

**DOI:** 10.3390/geriatrics9010012

**Published:** 2024-01-13

**Authors:** Alice Kit Ying Chan, Yiu Cheung Tsang, Eddie Hsiang-Hua Lai, Chun Hung Chu

**Affiliations:** 1 Faculty of Dentistry, The University of Hong Kong, Hong Kong 99907, China; dralice@hku.hk (A.K.Y.C.); elvist@hku.hk (Y.C.T.); 2 School of Dentistry, College of Medicine, National Taiwan University, Taipei 103247, Taiwan; 3 Department of Dentistry, National Taiwan University Hospital, Taipei 103247, Taiwan; 4 Department of Oral Health, Ministry of Health and Welfare, Taipei 103247, Taiwan

**Keywords:** older adults, elderly, tooth wear, oral health, prevention, fluoride, gerodontology, aging

## Abstract

Introduction: Tooth wear is a prevalent dental condition among older adults, leading to pain and adversely affecting aesthetics, functionality, and their overall quality of life. This review aims to update the information on tooth wear in older adults from the past five years and to provide guidance on the clinical management of tooth wear in older adults. Methods: A literature search was conducted in three electronic databases, Scopus, Pubmed, and Embase, for English publications from January 2019 to December 2023 on clinical studies with participants aged 65 or above on tooth wear. A total of 307 articles were retrieved and 14 articles were finally included as references for this study. Results: This review highlights the common causes of tooth wear and various risk factors, such as medical conditions, hyposalivation, dietary habits, oral hygiene practices, parafunctional habits, and occlusal factors, associated with tooth wear. It is crucial for oral health care professionals to diagnose and manage tooth wear at an early stage through a risk assessment and a clinical examination to avoid complex restorative procedures. Tooth wear management should prioritize prevention, aiming to control etiological and risk factors while employing non-restorative treatments. Restorative intervention, if indicated, should be simple, minimally invasive, and cost-effective. Tooth wear progression should be monitored regularly to determine if a further intervention is needed. Conclusion: Since the clinical studies on tooth wear in older adults over the past five years are limited and mainly cross-sectional, more interventional clinical studies are warranted to provide more clinical guidance on tooth wear management in older adults.

## 1. Introduction

Tooth wear is a common dental condition in the older adult population, which is categorized by the World Health Organization as the age group of 65 or above [1,2]. Tooth wear can be defined as the cumulative surface loss of mineralized tooth substance due to physical and chemo-physical processes not related to dental caries, resorption, or trauma [3]. Three mechanisms of tooth wear are dental attrition, dental erosion, and dental abrasion. Dental attrition is the physical loss of mineralized tooth substance caused by tooth-to-tooth contact [3]. Dental erosion is the chemical loss of mineralized tooth substance caused by exposure to acids not derived from oral bacteria [3]. Dental abrasion is the physical loss of mineralized tooth substance caused by objects other than teeth [3]. Tooth wear is associated with several risk factors, such as medical conditions, hyposalivation, dietary habits, oral hygiene habits, parafunctional habits, and occlusal factors [4]. Tooth wear is an age-related process, and the extent of it may vary from mild enamel loss to severe tooth substance loss with dentine exposure and pulpal involvement [5]. Age-related tooth wear can gradually be adapted by secondary dentine formation and dentoalveolar compensation, resulting in a change in the occlusal curves and planes [6,7]. Proximal tooth wear is attributed to the mesial drifting of the dentition. All these will affect the tooth alignment and occlusion and, hence, impact the aesthetics and function in older adults [7]. People will experience a certain degree of tooth wear during their life course without it affecting their health [6]. This age-related process is defined as physiological tooth wear [3]. However, when tooth wear progresses beyond the physiological level to the individual’s age and adaptation, it may induce symptoms and affect health and daily life [5]. This type of tooth wear is defined as pathological tooth wear and results in dentine hypersensitivity and pain; if left untreated, it may lead to tooth loss in older adults [3,8]. The affected teeth become flattened and shorter in length; they also look more yellowish with the underlying dentine exposed. Older adults may complain of poor aesthetics regarding the yellow and short teeth [8]. Older adults with severe tooth wear have a reduced occlusal vertical dimension, which lowers their maximum biting force and chewing efficiency [9]. These inevitably affect the oral-health-related quality of life in older adults [10,11].

Tooth wear should be managed first by making an accurate diagnosis and addressing the aetiological factors, followed by less invasive non-restorative treatments such as employing neutralizing or remineralizing agents to relieve symptoms and to halt the progression of tooth wear [12]. After addressing the underlying aetiological factors, restorative treatment may be considered for older adults with severe tooth wear to improve aesthetics, restore function, and provide the long-term preservation of dentition in older adults [12]. Restorative work for severe tooth wear is complicated and extensive. It involves providing multiple direct or indirect restorations with a new occlusion scheme and an occlusal vertical dimension for older adults [5]. Furthermore, restorative work requires multiple dental visits with an extended treatment time.

However, extensive restorative work for managing severe tooth wear is not feasible for older adults who are medically compromised, physically disabled, cognitively impaired, or financially disadvantaged [13]. Special precautions, such as anxiety control and blood pressure monitoring, are required for older adults who are medically compromised before they receive extensive dental treatment [14]. Those who are physically disabled may experience difficulties in arranging transportation and an escort to access the dental clinic for multiple visits [14]. Older adults who are cognitively impaired may have difficulties understanding the required procedure and adapting to the new restorations and occlusion [15]. The cost for extensive restorative work may not be affordable for those who are retired or financially disadvantaged [13]. In addition, the restorative work for managing tooth wear in older adults requires a substantial workforce and resources in our health care system.

Tooth wear is cumulative during a person’s life course, and its severity increases with age [16,17]. As the life expectancy increases and people retain their dentition longer, the prevalence and severity of tooth wear in older adults are expected to surge in the coming decade. The prevalence of tooth wear varies widely over the world, with a high prevalence in the older adults of some European countries [12,18,19]. More than two thirds of older adults have severe tooth wear in Greece and Belgium [16,20]. In China, almost all older adults exhibit signs of tooth wear with dentine exposure [17]. In Sudan, nearly 80% of older adults suffer from tooth wear, with almost 40% of them with dentine involvement and 14% with pulpal involvement [21]. The rising prevalence of tooth wear will inevitably have a significant effect on individual older adults, their caregivers, and health care systems.

The FDI World Dental Federation adopted a policy statement on tooth wear at the FDI General Assembly in Sydney in September 2023 [22]. This policy statement acknowledged the significant impacts of tooth wear on oral health [22]. Therefore, it is important for oral health care professionals to detect and manage tooth wear in older adults at an early stage. It not only enables the preservation of the natural dentition of older adults, but also avoids the necessity of complex and extensive restorative treatment. This review aims to update the information on tooth wear in older adults from the past five years and provide guidance on the clinical management of tooth wear in older adults.

## 2. Literature Search

This review is based on English publications identified in the Scopus, PubMed, and Embase databases on tooth wear in older adults from January 2019 to December 2023. The search was conducted using a combination of both MeSH words and non-MeSH words “tooth wear”, “tooth surface loss”, “aged”, “elderly”, and “older adults”. The last search was conducted on 20 December 2023. A total of 307 articles were retrieved. After removing the 137 duplicates, the titles and abstracts of 170 articles were screened for eligibility. Only clinical studies, cross-sectional studies, longitudinal studies, and clinical trials with participants aged 65 or above that investigated tooth wear were included for the full-text screening. Laboratory studies, case reports, case series, commentaries, protocols, literature reviews, and systematic reviews were excluded. Publications whose full texts could not be accessed were also excluded. Fourteen articles were finally included as the references to develop this study’s framework. Table 1 shows the MeSH and non-MeSH words used for the literature search and Table 2 presents the literature search and results in each database. Table 3 lists the included clinical studies on tooth wear in older adults.

## 3. Aetiology

Tooth wear is a multifactorial condition with interactions among the processes of attrition, abrasion, and erosion. The success of management depends on accurately identifying and controlling the causative agents. Attrition is the result of tooth-to-tooth contacts during normal function in chewing and grinding [7]. Abrasion is commonly caused by improper oral hygiene practices such as vigorous toothbrushing and using hard-bristled toothbrushes, abrasive toothpastes, and toothpicks [7]. Abrasion cavities can also be created by other parafunctional habits, such as opening hair pins with teeth or biting nails, pins, thread, a wind instrument, or other hard objects [7]. Erosion is induced by exposure to extrinsic and intrinsic acid [7]. The sources of extrinsic acid are diet, medicine/supplements, and a person’s environment/occupation. Teeth erode at a pH of 2.0 to 4.0, and hence, food/drink items with a pH lower than 4.0 are considered erosive to dentition [31]. 

Fruits play an important role in healthy aging, and older adults are always encouraged to increase their fruit or fruit juice consumption [32]. The daily consumption of food/drink such as citrus fruits, wine, fruit juice, carbonated drinks, and other foods containing vinegar leads to erosion [31]. Older adults who have chronic medical conditions or nutritional deficiencies may take medications or supplements [1,31]. Medications such as aspirin and supplements such as chewable vitamin C tablets are acidic in nature and can cause erosion [31]. Extrinsic acid can also come from the occupational or environmental exposure to certain chemicals or substances, such as acid fumes (sulfuric, hydrochloric, or nitric) to industrial workers or chlorinated water to swimmers [7]. However, it is not a common cause of erosive tooth wear [12]. Gastric acid is often the source of intrinsic acid for erosive tooth wear. The frequent regurgitation of gastric contents over an extended period can result in erosion [12]. Erosive tooth wear in older adults can result from some common gastrointestinal disorders, such as gastroesophageal reflux disease (GERD), a hiatal hernia, or duodenal or peptic ulcers [7,19]. Certain medications, such as levodopa for Parkinson’s disease and aminophylline for chronic obstructive pulmonary diseases, may cause nausea or vomiting and result in erosion [7].

## 4. Clinical Signs and Symptoms

The changes in the early stage of tooth wear are subtle and they are difficult to detect clinically. Different types of tooth wear vary in their clinical presentation. Attrition usually occurs on the occlusal and incisal surfaces and appears as a small polished wear facet on the cusp or marginal ridge or a slight flattening of the incisal edge [7]. The facets correspond to the functional movement of the dentition and are flat, sharp, bordered, and glossy [12]. Abrasion is most often found at the cervical area of the buccal surfaces of canines and premolars [7]. It appears as a rounded or V-shaped defect at the cementoenamel junction. Sometimes, it may be detected at the interproximal area created by the improper use of toothpicks or dental floss [7]. In contrast to abrasion, erosion affects the smooth surface area coronal to the cementoenamel junction, leaving an intact band of enamel at the gingival margin [3]. Erosion also causes the cupping of the cusps and the flattening of the occlusal surfaces [3]. An occlusal surface affected by erosion is matte in appearance, whereas those affected by attrition look shiny [3]. Restoration may protrude from the surrounding eroded surfaces [3]. 

When tooth wear progresses, it exposes the underlying dentine and the teeth look more yellow in colour. In the advanced stage, the teeth lose their entire occlusal morphology and contour and they become shorter in length [12]. The patient may experience dentine hypersensitivity or pain and they might complain of poor aesthetics due to generalized short and yellow dentition [5]. It is important for oral health care professionals to detect the early signs and symptoms of tooth wear and implement appropriate care to stop its progression and avoid further complicated treatment for older adults.

## 5. Risk Factors

**Figure 1:**
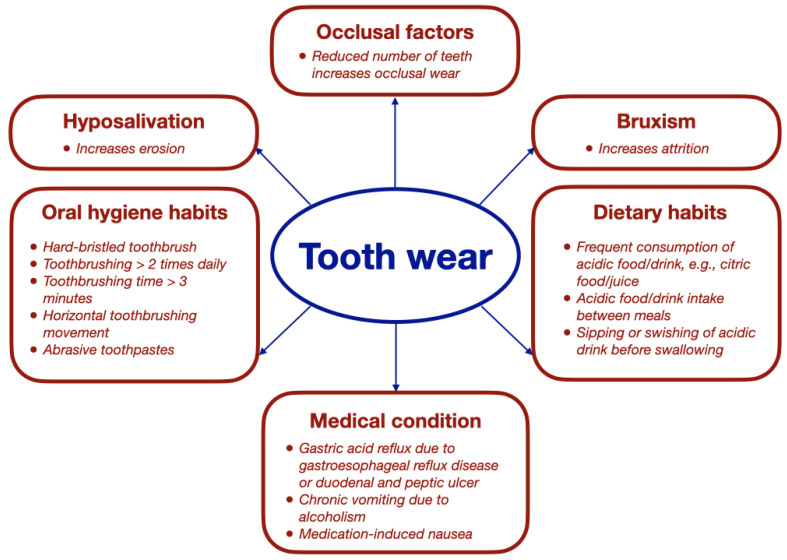
Risk factors associated with tooth wear.

Knowledge of risk factors helps oral health care professionals detect older adults who are at risk of tooth wear, allowing them to implement preventive measures at an early stage. Several risk factors have been associated with tooth wear. Figure 1 illustrates these risk factors.

### 5.1. Aging

Aging is reported as a risk factor of tooth wear, with an odds ratio of 3.9 [20]. The prevalence and severity of tooth wear increase as age increases. In China, the prevalence of tooth wear increased from 67.5% in adults to 100% in older adults [17]. The prevalence of severe tooth wear increased from 53% in Greek older adults aged between 60 and 70 to 79% in those over 70 years old [16]. 

### 5.2. Medical Conditions

Medical conditions such as gastroesophageal reflux disease, chronic vomiting, chronic alcoholism, and eating disorders increase the risk of erosive tooth wear in older adults at an odds ratio of 3.1 [20]. Individuals who presented with daily symptoms of gastroesophageal reflux disease had an increased risk of developing severe erosive tooth wear at an odds ratio of 3.8 [33]. There has been an increasing focus on the relationship between sleep apnoea and tooth wear; however, more studies are needed for further investigation [34]. A cross-sectional study observed that obesity was positively associated with tooth wear, yet this may have been partly related to their common risk factors, such as the consumption of sugar-sweetened acidic drinks [28].

### 5.3. Hyposalivation

Saliva plays an important role in erosive tooth wear through two mechanisms [35]. The first is the dilution and clearing of the acid in the oral cavity [35]. The second is the neutralization of the acid and lowering of the pH in the mouth because of saliva’s buffering components, such as bicarbonate, phosphate, and protein buffer [35,36]. A study showed that hyposalivation increased the severity of erosive tooth wear at an odds ratio of 3.8 [33]. Hyposalivation is a common oral condition in older adults, affecting one third of their population [1]. An increased age and chronic medical conditions such as diabetes contribute to hyposalivation [1]. Medications such as diuretics, anti-depressants, and anti-hypertensive, anti-Parkinsonian, and anti-retroviral drugs can induce hyposalivation [1,29]. Therefore, older adults with hyposalivation may have an increased risk of developing erosive tooth wear.

### 5.4. Dietary Habits

Erosive tooth wear has been strongly linked to dietary habits [31]. The frequency and timing of food intake is associated with the risk of erosion. The frequent consumption of acidic fruits is significantly associated with erosive tooth wear in older adults [37]. The timing of fruit intake is also crucial. Fruit intake between meals corresponds to an increased risk of tooth wear progression at an odds ratio of 3.6 when compared to fruit intake with meals [38]. Drinking habits are also important. Drinking acidic beverages through a straw can reduce the risk of erosion compared to drinking in sips over an extended period or swishing the beverage around the teeth [12]. Retaining the acidic drink in the mouth before swallowing increases the risk of erosion development and progression [12].

### 5.5. Oral Hygiene Habits

A toothbrushing frequency of more than twice a day, a toothbrushing duration of more than 3 min, and using a hard-bristled toothbrush, a horizontal toothbrushing movement, a high toothbrushing pressure, and abrasive toothpastes increases the likelihood of tooth wear [4]. A recent study showed that older adults with harmful toothbrushing habits had an increased risk of having non-carious cervical surface loss [27]. Some studies have suggested that toothbrushing soon after having an acidic food or drink may increase the prevalence of abrasion because the softened enamel may be more vulnerable when abraded [12]. To date, no studies have found any association between brushing after acid intake and tooth wear [4]. 

### 5.6. Bruxism

Bruxism is the non-functional gnashing, grinding, or clenching of teeth unconsciously while awake or during sleep, and it is believed to exaggerate the attrition process [7]. Most people with bruxism are not aware of this problem, but their family members alert them to it because of the noises they make at night. Most of the studies showing an association between sleep bruxism and occlusal wear are mainly based on self-reported bruxism [4]. Some have challenged the reliability of self-reporting bruxism and have suggested that polysomnography or electromyography should be used to confirm the association between sleep bruxism and tooth wear [4]. Therefore, the evidence on the association between sleep bruxism and tooth wear is still not solid [39].

### 5.7. Occlusal Factors

Occlusal factors have been proposed as one of the causes of non-carious cervical lesions [40]. The term “abfraction” is used to describe cervical tooth surface loss due to occlusal stress [40]. Presumably, an oblique occlusal force may induce tensile stress and disrupt the bonding between hydroxyapatite crystals, causing the separation of the enamel from dentine and, hence, the cervical lesions [40]. However, the current evidence does not support an association between occlusal factors and non-carious cervical lesions, and therefore, the use of the term “abfraction” is discouraged [3,40]. However, occlusal factors have been associated with the prevalence of attrition [4]. Studies have shown that dental arches with a lower number of remaining teeth have a higher prevalence of occlusal wear [4].

### 5.8. Risk Assessment

Tooth wear causes the irreversible loss of tooth substance, and the process is gradual and progressive in nature. The progression of tooth wear can be controlled or arrested by implementing appropriate preventive measures and interventions [8]. Therefore, the early detection of tooth wear is paramount to halting the damage and preventing the necessity of complex treatments for older adults [8]. This can be achieved by conducting a risk assessment using the patient’s history and detecting the early clinical signs of tooth wear [8]. Oral health care professionals should obtain a comprehensive history, including medical conditions, drug consumption, and a family and social history, from each older adult, and they should identify all the potential risk factors of tooth wear for the older adult [8]. Any medical conditions that may induce gastric acid reflux should be recorded in detail [8]. If in doubt, oral health care professionals should consult the patient’s medical practitioners [14]. Drugs that are acidic in nature or that may induce xerostomia, gastric reflux, or vomiting should all be identified [8]. Dietary habits can be assessed by asking older adults or their caregivers to record their daily intake of food, drink, and medication, specifying the amount, types, and time of each intake [8]. The person’s specific eating and drinking habits should also be recorded [12]. Oral hygiene habits, including the type of toothbrushing and toothpaste and the frequency and timing of toothbrushing, should also be noted. Oral health care professionals should ask older adults or their family members about any daytime or sleep bruxism or destructive biting habits. Older adults who show signs and symptoms of hyposalivation, who have received head and neck radiation, or who have medical conditions or take medications that affect salivary flow should have a saliva analysis in terms of the flow rate and buffering capacity [8]. A thorough history-taking can help highlight older adults who are at risk of tooth wear for education and prevention. The Special Interest Working Group on Tooth Surface Loss/Erosion of the European Association of Dental Public Health generated a new risk assessment tool for erosive tooth wear, the Erosive Wear Assessment of Risk (EWAR) tool, which consists of a risk factor questionnaire and a saliva secretion evaluation [41]. This new tool, when combined with a clinical index, can be used for erosive tooth wear risk assessments for chairside use [41]. Additionally, it will provide guidance if the monitoring of tooth wear or an intervention is indicated [5].

## 6. Tooth Wear Management in Older Adults

Tooth wear management in older adults should start at prevention. Prevention aims to reduce or halt the progression of tooth wear. It can be achieved by controlling all potential aetiological and risk factors and by using non-restorative treatment [12]. 

### 6.1. Control of All Potential Aetiological and Risk Factors

All potential aetiological and risk factors should be identified using a thorough history-taking, an oral examination, and, if indicated, a special investigation such as a salivary test. Oral health care professionals should inform older adults and their caregivers about the findings collected and their responsibility for controlling these risk factors. Tailored advice and prevention should be given accordingly. If intrinsic acid is indicated, older adults should be referred to a specialist for a further consultation and proper management; if extrinsic acid is suggested, dietary advice should be given. Most dietary acids are considered weak acids and have a low erosive potential to cause dental erosion. Food or drink items with a low pH value and a high buffering capacity have a high erosive potential. Oral health care professionals should advise older adults to avoid or reduce their intake frequency of food or drink with a high erosive potential, such as citric fruit juices (orange and lemon) and soft drinks, and consume food or drink with a low pH and a higher content of calcium and phosphate, such as milk and yoghurt [4,8]. 

Older adults should also avoid eating and drinking habits that may extend the contact time between acid and their teeth [12]. If older adults use medications with a high erosive potential, oral health care professionals should discuss with their physician any alternative options [8]. Older adults with hyposalivation can use saliva substitutes based on formulations with calcium phosphate and fluoride to provide protection against erosion [8]. Older adults with bruxism should be informed about its potential to cause tooth wear and should wear an occlusal splint to prevent tooth wear. Older adults should receive oral hygiene instructions on their choice of toothbrush and toothpaste, proper toothbrushing techniques, and appropriate toothbrushing frequency and time.

### 6.2. Non-Restorative Treatment

Some protective agents have been suggested to reduce or prevent tooth wear progression. The role of fluoride in caries prevention has been well proven, and researchers have found positive results for the use of fluoride in preventing dental erosion [42]. One study proposed that fluoride could prevent or reduce tooth wear by forming acid-resistant fluorapatite and enhancing remineralization [42]. However, fluoride’s effect was only limited to the surface layer of the softened enamel and the protective effect was small [42]. Some in vitro studies have also demonstrated positive results for using products containing polyvalent metal ions (tin or titanium) or products with added amino acids, peptides, or proteins in preventing dental erosion [42]. Fluoridated products containing metal cations, such as stannous was found to be a better option than other fluoride solutions in controlling dental erosion [43]. 

Some researchers considered that fluoridated products containing stannous formed tin deposition on tooth surfaces and incorporated into the near-surface layer of enamel [42]. The tin-rich deposition was more resistant to an acid attack, and hence, it prevented the underlying tooth substance from dental erosion [42]. An in vitro study found that the combined use of stannous fluoride-containing mouth rinses and toothpastes can increase their protective effect against dental erosion [43]. Toothpastes with added polymers such as sodium hexametaphosphate and chitosan, which adsorb to dental surfaces and create an additional protective layer, may protect against dental erosion [8]. However, all these results are from in vitro studies, and more in situ/in vivo research will be needed to confirm the role of these products in preventing dental erosion [42]. Oral health care professionals can also apply desensitizing agents for older adults who present with dentine hypersensitivity due to tooth wear [44]. A recent trial showed that 38% silver diamine fluoride was effective in reducing dentine hypersensitivity in older adults [45]. Applying a resin sealant or bonding agent may also reduce tooth wear progression and relieve dentine hypersensitivity in the short term [8,12].

### 6.3. Restorative Management

Not all older adults with tooth wear need restorative treatment. Tooth wear is an age-related change and may not necessarily affect health [6]. However, when it advances and causes pain or discomfort, impairs function, or deteriorates aesthetics, it may warrant restorative treatment [5]. This type of treatment must be performed after or in conjunction with preventive measures. Without addressing the aetiological and risk factors, all the restorative work will only be short-lived.

Worn dentition can be restored using various methods, including direct or indirect techniques and using different materials, such as composites, ceramics, and metals [5]. A direct technique using composite restorations is simpler, less invasive, and more cost-effective than an indirect technique [5]. A longitudinal study showed that restoration using either glass-ceramics or resin composites can be a choice for permanent use in patients with severe tooth wear [30]. As a rule, restorative treatment for older adults should be simple and conservative due to their underlying medical, physical, and cognitive condition. For older adults with tooth loss and tooth wear, an overlay denture can be considered to replace missing teeth and restore worn dentition together as a simple, conservative, and less costly alternative. However, it depends on the number of missing teeth and the degree of tooth wear.

### 6.4. Monitoring Tooth Wear

Older adults exhibiting signs of tooth wear should receive regular monitoring for tooth wear progression. This can help confirm the aetiology, evaluate the effectiveness of preventive measures, and determine the need for further interventions [8]. Tooth wear progression can be monitored using clinical signs such as dentine hypersensitivity, a dull and frosty appearance, or the absence of staining over a lesion [12]. The presence and severity of tooth wear can be recorded according to a visual examination using clinical indices. The recommended index is the Basic Erosive Wear Examination (BEWE) index, which assesses the most severe lesion in each sextant using a 4-score grading system (score of 0: no erosion; 1: loss of enamel surface texture; 2: hard-tissue loss < 50% of the surface area; and 3: hard-tissue loss ≥ 50% of the surface area) [8,12]. The BEWE index also links the scoring to clinical management [12]. Longitudinal study models and standardized photographs can also be used for monitoring the progression [8]. With intraoral scanning, tooth wear progression may be measured quantitatively for future comparisons [46].

This review was subject to several limitations. There may have been a selection bias because the literature search was not conducted using a systematic approach. Since the number of clinical studies investigating tooth wear in older adults was limited, this review included clinical studies conducted in both adult and older adult groups. Therefore, the results may not be specific to older adults. Most of the clinical studies included were cross-sectional studies, and the evidence level was not strong. This review provides some insights for future research. Few clinical studies were conducted on tooth wear in older adults over the past five years, and most were cross-sectional studies focusing on prevalence and risk factors. There should be more interventional clinical studies on tooth wear management in older adults to provide more guidance in clinical practice.

## 7. Conclusions

Tooth wear is a common dental problem in older adults. Severe tooth wear requires extensive and complicated restorative work in older adults. It is important for oral health care professionals to identify tooth wear in an early stage and to implement prevention and non-restorative treatment in older adults. This can halt tooth wear progression and avoid the necessity for extensive, invasive, and expensive restorative treatments in older adults. Since the clinical studies on tooth wear in older adults over the past five years are limited and mainly cross-sectional, more interventional clinical studies are warranted to provide more clinical guidance on tooth wear management in older adults. 

## Figures and Tables

**Table 1:** MeSH and non-MeSH words used for literature search.

MeSH Words	Non-MeSH Words
tooth wear; aged	tooth surface loss; elderly; older adults

**Table 2:** Literature search and results in each database.

Database	Search Terms	Results
Scopus	((TITLE-ABS-KEY (“tooth wear”) OR TITLE-ABS-KEY (“tooth surface loss”))) AND ((TITLE-ABS-KEY (“aged”) OR TITLE-ABS-KEY (“elderly”) OR TITLE-ABS-KEY (“older adults”)))	160
Pubmed	(“tooth wear” OR “tooth surface loss”) AND (“aged” OR “elderly” OR “older adults”)	109
Embase	“tooh wear” “tooth surface loss”.mp. 1 or 2 “aged” “elderly”.mp. “older adults”.mp. 4 or 5 or 6 3 and 7	38

**Table 3:** The included clinical studies on tooth wear in older adults.

Study Type (Author, Year)	Title	Findings
*Prevalence*		
Cross-sectional study (Salih et al., 2022) [21]	“Oral health status and associated factors among Sudanese older adults: A cross-sectional study”	Tooth wear was found in 46.2% of the older adult participants.
Cross-sectional study (Theodoridis et al., 2022) [18]	“Tooth wear epidemiology and its associated periodontal health and sociodemographic factors in a cluster of senior citizens in Northern Greece”	Almost all senior citizens in Northern Greece presented with signs of tooth wear.
Cross-sectional study (Kitasako et al., 2021) [23]	“The prevalence of non-carious cervical lesions (NCCLs) with or without erosive etiological factors among adults of different ages in Tokyo”	NCCL distribution increased with age, and erosive dietary risk factors might affect the incidence of NCCLs for elders.
Cross-sectional study (Stangvaltaite-Mouhat et al., 2020) [19]	“Erosive tooth wear among adults in Lithuania: a cross-sectional national oral health study”	Erosive tooth wear was found in 63% of older adults. Males, residency in peri-urban/rural areas, older age, and presence of acid reflux were associated with higher odds of erosive tooth wear.
Cross-sectional study (Kaklamanos et al., 2020) [16]	“Tooth wear in a sample of community-dwelling elderly Greeks”	Severe wear was more prevalent with advanced age, more extensive occlusal/incisal surfaces in males, and those with fewer than 20 remaining teeth.
*Impact of tooth wear on older adults*		
Cross-sectional study (Kanaan et al., 2022) [24]	“Tooth wear and oral-health-related quality of life in dentate adults”	Distal and proximal indicators for tooth wear were mediators for impaired oral-health-related quality of life and treatment needs.
Cross-sectional study (Mehta et al., 2020) [11]	“An investigation into the impact of tooth wear on the oral health related quality of life amongst adult dental patients in the United Kingdom, Malta and Australia”	Higher levels of tooth wear were significantly associated with a deteriorating oral-health-related quality of life amongst the participants.
Cross-sectional study (Levartovsky et al., 2020) [25]	“The effect of tooth wear, age and sex on facial height assessed by soft tissue analysis”	The proportion of the upper to lower facial segments was constant and was not affected by age or tooth wear.
*Risk factors*		
Cross-sectional study (Kanaan et al., 2022) [20]	“Non-biological and biological risk indicators for tooth wear outcomes in adults”	Medical conditions and consumption of acidic beverages were moderate and weak risk indicators, respectively, for tooth wear.
Cross-sectional study (Kalsi et al., 2021) [26]	“Quality of life and other psychological factors in patients with tooth wear”	Increased severity of tooth wear was correlated with older age and worse generic and condition-specific quality of life.
Cross-sectional study (Penoni et al., 2021) [27]	“Factors associated with noncarious cervical lesions in different age ranges: a cross-sectional study”	Elderly people with harmful toothbrushing habits had a greater mean NCCL.
Cross-sectional study (Kamal et al., 2020) [28]	“Obesity and tooth wear among American adults: the role of sugar-sweetened acidic drinks”	Obesity was positively associated with tooth wear and this association was only partially accounted for by the consumption of sugar-sweetened acidic drinks, a common risk factor for both conditions.
Cross-sectional study (Sehgal et al., 2019) [29]	“Tooth wear in patients treated with HIV anti-retroviral therapy”	HIV +ve patients on anti-retroviral therapy had significant tooth wear.
*Management*		
Longitudinal study (Edelhoff et al., 2023) [30]	“Pressable lithium disilicate ceramic versus CAD/CAM resin composite restorations in patients with moderate to severe tooth wear: Clinical observations up to 13 years”	Single-tooth restorations of both materials can be recommended for permanent use in patients with severe tooth wear.

## References

[1] Chan A.K.Y., Tamrakar M., Jiang C.M., Lo E.C.M., Leung K.C.M., Chu C.-H. (2021). Common Medical and Dental Problems of Older Adults: A Narrative Review. Geriatrics.

[2] World Health Organization (2001). Men, Ageing and Health: Achieving Health Across the Life Span.

[3] Schlueter N., Amaechi B.T., Bartlett D., Buzalaf M.A.R., Carvalho T.S., Ganss C., Hara A.T., Huysmans M.-C.D., Lussi A., Moazzez R. (2020). Terminology of Erosive Tooth Wear: Consensus Report of a Workshop Organized by the ORCA and the Cariology Research Group of the IADR. Caries Res..

[4] Oudkerk J., Grenade C., Davarpanah A., Vanheusden A., Vandenput S., Mainjot A.K. (2023). Risk factors of tooth wear in permanent dentition: A scoping review. J. Oral Rehabil..

[5] Loomans B., Opdam N., Attin T., Bartlett D., Edelhoff D., Frankenberger R., Benic G., Ramseyer S., Wetselaar P., Sterenborg B. (2017). Severe Tooth Wear: European Consensus Statement on Management Guidelines. J. Adhes. Dent..

[6] Bartlett D., O’Toole S. (2020). Tooth Wear: Best Evidence Consensus Statement. J. Prosthodont..

[7] Hattab F.N., Yassin O.M. (2000). Etiology and diagnosis of tooth wear: A literature review and presentation of selected cases. Int. J. Prosthodont..

[8] Carvalho J.C., Scaramucci T., Aimée N.R., Mestrinho H.D., Hara A.T. (2018). Early diagnosis and daily practice management of erosive tooth wear lesions. Br. Dent. J..

[9] Arima T., Takeuchi T., Honda K., Tomonaga A., Tanosoto T., Ohata N., Svensson P. (2013). Effects of interocclusal distance on bite force and masseter EMG in healthy participants. J. Oral Rehabil..

[10] Papagianni C.E., van der Meulen M.J., Naeije M., Lobbezoo F. (2013). Oral health-related quality of life in patients with tooth wear. J. Oral Rehabil..

[11] Mehta S., Loomans B., Banerji S., Bronkhorst E., Bartlett D. (2020). An investigation into the impact of tooth wear on the oral health related quality of life amongst adult dental patients in the United Kingdom, Malta and Australia. J. Dent..

[12] Carvalho T.S., Colon P., Ganss C., Huysmans M.C., Lussi A., Schlueter N., Schmalz G., Shellis R.P., Tveit A.B., Wiegand A. (2015). Consensus report of the European Federation of Conservative Dentistry: Erosive tooth wear—Diagnosis and management. Clin. Oral Investig..

[13] Chan A.K.Y., Tamrakar M., Leung K.C.M., Jiang C.M., Lo E.C.M., Chu C.-H. (2021). Oral Health Care of Older Adults in Hong Kong. Geriatrics.

[14] Chan A.K.Y., Tsang Y.C., Jiang C.M., Leung K.C.M., Lo E.C.M., Chu C.H. (2023). Integration of Oral Health into General Health Services for Older Adults. Geriatrics.

[15] Schaper S., Meyer-Rötz S., Bartels C., Wiltfang J., Rödig T., Schott B.H., Belz M. (2021). Dental Care of Patients with Dementia: A Survey on Practice Equipment, Training, and Dental Treatment. Front. Oral Health.

[16] Kaklamanos E.G., Menexes G., Makrygiannakis M.A., Topitsoglou V., Kalfas S. (2020). Tooth Wear in a Sample of Community-Dwelling Elderly Greeks. Oral Health Prev. Dent..

[17] Wei Z., Du Y., Zhang J., Tai B., Du M., Jiang H. (2016). Prevalence and Indicators of Tooth Wear among Chinese Adults. PLoS ONE.

[18] Theodoridis C., Menexes G., Topitsoglou V., Kalfas S. (2022). Tooth Wear Epidemiology and Its Associated Periodontal Health and Sociodemographic Factors in a Cluster of Senior Citizens in Northern Greece. Dent. J..

[19] Stangvaltaite-Mouhat L., Pūrienė A., Stankeviciene I., Aleksejūnienė J. (2020). Erosive Tooth Wear among Adults in Lithuania: A Cross-Sectional National Oral Health Study. Caries Res..

[20] Kanaan M., Brabant A., Eckert G.J., Hara A.T., Carvalho J.C. (2022). Non-Biological and Biological Risk Indicators for Tooth Wear Outcomes in Adults. Caries Res..

[21] Salih M.A., Ali R.W., Nasir E.F. (2022). Oral health status and associated factors among Sudanese older adults: A cross-sectional study. Gerodontology.

[22] Federation FWD FDI Policy Statement Tooth Wear 2023. https://www.fdiworlddental.org/sites/default/files/2023-10/4.%20EN_FDPS3_Tooth%20wear.pdf.

[23] Kitasako Y., Ikeda M., Takagaki T., Burrow M.F., Tagami J. (2021). The prevalence of non-carious cervical lesions (NCCLs) with or without erosive etiological factors among adults of different ages in Tokyo. Clin. Oral Investig..

[24] Kanaan M., Brabant A., Eckert G.J., Hara A.T., Carvalho J.C. (2022). Tooth wear and oral-health-related quality of life in dentate adults. J. Dent..

[25] Levartovsky S., Aharonov O., Perlman A.E., Winocur E., Sarig R. (2020). The effect of tooth wear, age and sex on facial height assessed by soft tissue analysis. J. Oral Rehabil..

[26] Kalsi H., Khan A., Bomfim D., Tsakos G., McDonald A.V., Rodriguez J.M. (2021). Quality of life and other psychological factors in patients with tooth wear. Br. Dent. J..

[27] Penoni D.C., Miranda M.E.d.S.N.G., Sader F., Vettore M.V., Leão A.T.T. (2021). Factors Associated with Noncarious Cervical Lesions in Different Age Ranges: A Cross-sectional Study. Eur. J. Dent..

[28] Kamal Y., O’toole S., Bernabé E. (2020). Obesity and tooth wear among American adults: The role of sugar-sweetened acidic drinks. Clin. Oral Investig..

[29] Sehgal H.S., Kohli R., Pham E., Beck G.E., Anderson J.R. (2019). Tooth wear in patients treated with HIV anti-retroviral therapy. BMC Oral Health.

[30] Edelhoff D., Erdelt K., Stawarczyk B., Liebermann A. (2023). Pressable lithium disilicate ceramic versus CAD/CAM resin composite restorations in patients with moderate to severe tooth wear: Clinical observations up to 13 years. J. Esthet. Restor. Dent..

[31] Chan A.K.Y., Tsang Y.C., Jiang C.M., Leung K.C.M., Lo E.C.M., Chu C.H. (2023). Diet, Nutrition, and Oral Health in Older Adults: A Review of the Literature. Dent. J..

[32] Dominguez L.J., Veronese N., Baiamonte E., Guarrera M., Parisi A., Ruffolo C., Tagliaferri F., Barbagallo M. (2022). Healthy Aging and Dietary Patterns. Nutrients.

[33] Alaraudanjoki V., Laitala M.-L., Tjäderhane L., Pesonen P., Lussi A., Ronkainen J., Anttonen V. (2016). Influence of Intrinsic Factors on Erosive Tooth Wear in a Large-Scale Epidemiological Study. Caries Res..

[34] Durán-Cantolla J., Alkhraisat M.H., Martínez-Null C., Aguirre J.J., Guinea E.R., Anitua E. (2015). Frequency of Obstructive Sleep Apnea Syndrome in Dental Patients with Tooth Wear. Sleep Med..

[35] Moazzez R., Bartlett D., Anggiansah A. (2004). Dental erosion, gastro-oesophageal reflux disease and saliva: How are they related?. J. Dent..

[36] Bardow A., Moe D., Nyvad B., Nauntofte B. (2000). The buffer capacity and buffer systems of human whole saliva measured without loss of CO_2_. Arch. Oral Biol..

[37] Kitasako Y., Sasaki Y., Takagaki T., Sadr A., Tagami J. (2015). Age-specific prevalence of erosive tooth wear by acidic diet and gastroesophageal reflux in Japan. J. Dent..

[38] O’toole S., Bernabé E., Moazzez R., Bartlett D. (2017). Timing of dietary acid intake and erosive tooth wear: A case-control study. J. Dent..

[39] Kapagiannidou D., Koutris M., Wetselaar P., Visscher C.M., van der Zaag J., Lobbezoo F. (2021). Association between polysomnographic parameters of sleep bruxism and attrition-type tooth wear. J. Oral Rehabil..

[40] Silva A.G., Martins C.C., Zina L.G., Moreira A.N., Paiva S.M., Pordeus I.A., Magalhães C.S. (2013). The association between occlusal factors and noncarious cervical lesions: A systematic review. J. Dent..

[41] Margaritis V., Alaraudanjoki V., Laitala M.-L., Anttonen V., Bors A., Szekely M., Alifragki P., Jász M., Berze I., Hermann P. (2021). Multicenter study to develop and validate a risk assessment tool as part of composite scoring system for erosive tooth wear. Clin. Oral Investig..

[42] Lussi A., Carvalho T.S. (2015). The Future of Fluorides and Other Protective Agents in Erosion Prevention. Caries Res..

[43] Jiemkim A., Tharapiwattananon T., Songsiripradubboon S. (2023). Combined use of stannous fluoride-containing mouth rinse and toothpaste prevents enamel erosion in vitro. Clin. Oral Investig..

[44] Chan A.K.Y., Tamrakar M., Jiang C.M., Tsang Y.C., Leung K.C.M., Chu C.H. (2022). Effectiveness of 38% Silver Diamine Fluoride in Reducing Dentine Hypersensitivity on Exposed Root Surface in Older Chinese Adults: Study Protocol for a Randomised Double-Blind Study. Dent. J..

[45] Chan A.K.Y., Tsang Y.C., Jiang C.M., Leung K.C.M., Lo E.C.M., Chu C.H. (2023). Treating hypersensitivity in older adults with silver diamine fluoride: A randomised clinical trial. J. Dent..

[46] O’toole S., Marro F., Loomans B.A.C., Mehta S.B. (2023). Monitoring of erosive tooth wear: What to use and when to use it. Br. Dent. J..

